# Awe and the Experience of the Sublime: A Complex Relationship

**DOI:** 10.3389/fpsyg.2020.01340

**Published:** 2020-06-16

**Authors:** Margherita Arcangeli, Marco Sperduti, Amélie Jacquot, Pascale Piolino, Jérôme Dokic

**Affiliations:** ^1^École des Hautes Études en Sciences Sociales, Paris, France; ^2^Institut Jean Nicod, UMR 8129, École Normale Supérieure, Paris, France; ^3^Laboratoire Mémoire, Cerveau et Cognition, URP 7536, Institut de Psychologie, Université de Paris, Boulogne-Billancourt, France

**Keywords:** awe, positive awe, threat-based awe, sublime, aesthetic experience, fear, admiration

## Abstract

Awe seems to be a complex emotion or emotional construct characterized by a mix of positive (contentment, happiness), and negative affective components (fear and a sense of being smaller, humbler or insignificant). It is striking that the elicitors of awe correspond closely to what philosophical aesthetics, and especially Burke and Kant, have called “the sublime.” As a matter of fact, awe is almost absent from the philosophical agenda, while there are very few studies on the experience of the sublime as such in the psychological literature. The aim of this paper is to throw light on the complex relationship between awe (as understood by psychologists) and the experience of the sublime (as discussed by philosophers). We distinguish seven ways of conceiving this relationship and highlight those that seem more promising to us. Once we have a clearer picture of how awe and the experience of the sublime are related, we can use it to enhance collaboration between these domains. We would be able to use empirical results about awe in a philosophical analysis of the experience of the sublime, which in turn can help us to design novel experimental hypotheses about the contexts in which we experience awe.

## Introduction

Since Keltner and Haidt (2003)’s seminal paper, psychologists have become increasingly interested in *awe*, an affective experience which is difficult to explain within the traditional dichotomy between positive and negative emotions.^1^ It is widely acknowledged that experiences of awe produce in general positive outcomes contributing to mental health (increased pro-social behavior, life-satisfaction and meaning of life – see, e.g., Rudd et al., 2012; Piff et al., 2015), and indeed most psychological studies have investigated awe as a positive emotion (see, e.g., Griskevicius et al., 2010; Campos et al., 2013; Shiota et al., 2017). However, although awe can be seen as having an *overall* positive valence, it has a negative flavor (Chirico et al., 2016, 2017). Awe seems to be a complex emotion or emotional construct characterized by a mix of positive (contentment, happiness), and negative affective components (fear and a sense of being smaller, humbler or insignificant). It is interesting to notice that beyond English “awe” is often captured by a combination of positive and negative terms meaning something like “fear mixed with admiration” (“timore reverenziale,” “effroi mêlé d’admiration,” “*Ehrfurcht*”, “
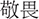
”).

Despite the protean nature of awe, it has been suggested that it is a *basic emotion* (Ekman, 1992), even having a distinctive facial expression which involves a particular pattern combining the gaze looking upward, the mouth open slightly, and slightly oblique eyebrows (Shiota et al., 2003). There is currently no consensus about these claims in the psychological literature, which, however, has found convergence points. Most studies, influenced by Keltner and Haidt (2003), have taken vastness and need for accommodation to be the prototypical appraisal themes of awe, which is thus defined as a strong emotional response to (physical or metaphorical) grand stimuli needing new conceptual/perceptual resources.

Interestingly, the description of awe in psychology matches well an aesthetic experience widely discussed in the philosophical literature, which has to do with the sublime. Here is a telling excerpt by Thomas Mann’s *The Magic Mountain* describing this kind of experience:

But if there was something roguish and fantastic about the immediate vicinity through which you laboriously made your way, the towering statues of snow-clad Alps, gazing down from the distance, awakened in you feelings of the sublime and holy (Mann, 1924/1996, p. 462).

Huge and steep mountains, starry night skies, waterfalls, grand canyons, deserts, thunderstorms are all examples of grand stimuli triggering experiences of the sublime. This type of experience arises when we are confronted with an overwhelming vastness or power and nature offers paradigmatic examples of such a grandeur.

Like awe, the experience of the sublime has an ambivalent valence (Brady, 2013). As an aesthetic experience, the experience of the sublime has an overall positive valence (Arcangeli et al., 2019), even though it also involves a negative affective evaluation of the world, something like terror (Burke, 1759), fear (Kant, 1790/2000) or a feeling of self-negation (Cochrane, 2012). Keltner and Haidt (2003) explicitly tie the concept of awe to the philosophical concept of the sublime, and see an analogy between the aspects they take awe to have (i.e., vastness and need for accommodation) and power and obscurity (as being difficult to grasp by intellect) in Burke’s seminal analysis of the sublime.

These considerations suggest that what psychologists call “awe” is what philosophers call “experience of the sublime.” Do we really have here one type of experience (multifaceted though it is) only, which has simply been labeled differently in different disciplines?

As a matter of fact, it is striking that awe is almost absent from the philosophical agenda, while there are very few studies on the experience of the sublime as such in the psychological literature (see, e.g., Eskine et al., 2012; Ishizu and Zeki, 2014; Hur et al., 2018). The aim of this paper is to throw light on the relationship between awe (as understood by psychologists) and the experience of the sublime (as discussed by philosophers). Once we have a clearer picture of how awe and the experience of the sublime are related, we can use it to enhance collaboration between these domains. We would be able to use empirical results about awe in a philosophical analysis of the experience of the sublime, which in turn can help us to design novel experimental hypotheses about the contexts in which we experience awe.

Although a terminological equivalence might recommend itself as the simplest, our goal is to show that alternative explanations of the relationship between awe and the experience of the sublime are worth exploring, opening up new paths of interdisciplinary enquiry. More precisely, through a careful analysis of the extant philosophical and psychological literature, we will sort out seven possible ways in which awe and the experience of the sublime connect. Some of them are less plausible than others and have been simply hinted at in passing by some authors. Accordingly, we will give more space to the most plausible views. In conclusion, we will briefly indicate what is in our view the most promising path to understand the complex relationship between awe and the experience of the sublime (henceforth ES).

## Seven Views on Awe and the Experience of the Sublime

At least seven views of the relationship between awe and the ES can be envisaged:

A. Awe and ES are the same type of experience.
B. Awe is an ingredient of ES.
C. ES is an ingredient of awe.
D. ES is a species of awe.
E. Awe is a species of ES.
F. Awe and ES share only a proper part.
G. Awe and ES are unrelated to each other.

Let us expand on each of these views in turn.

### The Equivalence Between Awe and the Experience of the Sublime

Option A is the equivalence view suggested by Keltner and Haidt (2003), although their more detailed view is that ES is awe with some additional “peripheral or flavoring” features, such as (experience of) beauty (see option D below). They seem to be followed by Fingerhut and Prinz (2018), who picture awe as intense wonder, and thus the sublime as a species of beauty. Some philosophers too seem to opt for option A. For instance, Brady (2013) writes: “It might be argued that the sublime is a relic best left alone, perhaps better replaced with a concept carrying less weighty historical and metaphysical baggage, such as ‘awe’ or ‘grandeur”’ (p. 2). In a similar vein, McShane (2013) notes: “The concept of the sublime as it has been discussed in philosophy (though not in literary criticism) from about the mid-eighteenth century onward I take to be the same concept as awe. Many other commentators seem to agree on this point; Burke’s and Kant’s analyses of the sublime are often discussed in analyses of the nature of awe” (p. 756, fn 34).

Option A entails that all the objects of ES are awe-inspiring. The latter claim is certainly plausible, which already enables us to exclude option G (i.e., that awe and ES have nothing in common). However, it is not clear that all awe-inspiring objects are also objects of ES or, for that matter, of any aesthetic experience at all. For instance, our awe of Mother Teresa’s compassion is arguably not aesthetic (McShane, 2013). Therefore, option A does not seem to be sustainable.

### Is Awe an Ingredient of the Experience of the Sublime or Vice-Versa?

Option B pictures awe as being an ingredient (either a causal determinant or a proper part) of ES. Brady (2013) herself gives voice to this option when she describes ES as a mixed feeling, “with certain negative feelings (*awe*, terror, etc.) felt alongside positive ones (exaltation, admiration)” (p. 40, our italics). She suggests that both Kant and Herder hold this view. Commenting on Herder, Zuckert (2003) writes: “[T]he viewer of sublime architecture such as St. Peter’s has a progressive experience: she approaches with a feeling of awe, enters and appreciates the decoration and elaboration, and then absorbs and is absorbed by the whole” (p. 220).

These philosophical observations hint at the idea that awe somehow captures the negative component of ES. This seems to be in contrast with what most psychologists advance, namely that awe is a positive emotion (see the Introduction). It should be noted, however, that some studies suggest the existence of two sorts of awe experiences, a positive and a negative one, that can be distinguished along several dimensions (subjective experience, physiological correlates and consequences on well-being).

A study by Piff et al. (2015) investigated two awe conditions, a positive and a negative one (elicited by videos about either non-threatening or threatening natural phenomena). They reported that both awe conditions, compared to the control condition, equally produced higher level of awe, and an increased sense of being diminished in the presence of something greater than us. By contrast, only negative awe produced increased negative emotions (e.g., anxiety, fear, and nervousness). Similar results were reported by Rivera et al. (2019). In the same vein, Sawada and Nomura (2020) showed that positive and negative awe-eliciting videos were rated more awe-inspiring, compared to a control condition, and increased happiness and anxiety ratings, respectively. This distinction is supported by a further work asking participants to describe a memorable awe experience and to report the elicitors, emotions and appraisals related to it (Gordon et al., 2017). Participants describing positive and threat-based awe experiences reported comparable levels of awe, but greater levels of fear were associated with the second kind of experience only.

Therefore, option B might be supported by claiming that, at least, negative (or threat-based) awe is an ingredient of ES. This view seems to be suggested in the psychological literature by Ishizu and Zeki (2014), who claim that ES “is a distinct cognitive-emotional complex” involving many components, awe included (which they associate with fear and horror), “but is distinct from each individually, i.e., that the whole is other than the parts” (p. 6).

Distinguishing between a positive and a negative type of awe can also be used in support of option C – i.e., the view that ES should be seen as an ingredient of awe. In philosophy this option has been suggested for instance by Kearney (1988) who, commenting on Kant, writes that “the sublime experience of overwhelming super-abundance produces a sense of ‘awe”’ (p. 175). Some psychologists, based on philosophical theories assigning a pivotal role to fear and terror in ES such that of Kant (see the Introduction), draw a parallel between the negative species of awe and ES (Gordon et al., 2017), which suggests that the latter is the negative ingredient (either a causal determinant or a proper part) of awe. This view, however, is based on the assumption that ES is mainly associated with strong negative emotions, especially fear. Few empirical studies have tried to investigate this subject. Eskine et al. (2012) reported that fear induction, but not induction of happiness or of general physiological arousal, can increase sublime ratings of pictorial abstract artworks. In another study sublimity ratings of photographs depicting natural scenarios were correlated with ratings of fear, but not with ratings of happiness (Hur et al., 2018). These data seem to suggest that ES is associated with fear (see also Chirico and Yaden, 2018), yet this is a questionable hypothesis, which has been nuanced by other works. It should be noted that Hur et al. (2018) themselves did not find any physiological evidence (from facial electromyography) linking sublimity ratings with physiological markers of fear. In one neuroimaging study on ES, Ishizu and Zeki (2014) reported that sublimity ratings of pictures of nature positively correlated with ratings of pleasantness. Moreover, although in line with philosophical treatment of ES they expected to find activation in brain areas classically associated with the experience of fear and threat such as the amygdala and the insula, their results did not show any such activity. Pelowski et al. (2019) investigated the cognitive-affective profile of ES in a large sample. They reported that the vast majority of reports (90.8%) could be classified under one category associated with positive emotions (e.g., pleasure). They also found a second statistically significant cluster associated with higher level of negative emotions, but this class was quite rare and was also associated with lower ratings of sublimity. It seems, thus, that the prototypical ES would be rather a positive experience (as suggested see the Introduction).

Taken together these findings show that there is not a clear-cut association between ES and either positive or negative emotions, and that probably, following Pelowski et al. (2019)’s suggestion, a positive and a negative variant of ES might exist. This would weaken the idea that the sublime is the dark side of awe, and more generally it puts pressure on option C (as well as on a specific reading of option D, as it will be made clear shortly).

### Is the Experience of the Sublime a Species of Awe or Vice-Versa?

The distinction made within the experimental literature between two sorts of awe can motivate a more ontologically demanding view than C, namely option D. While according to the former ES is an ingredient of awe, the latter claims that ES is a *species* of awe. Therefore, depending on how we interpret what sets apart positive and negative awe (i.e., whether they are two aspects of the same species or two species, possibly belonging to the same genus), we can end up with the view that ES coincides with the negative species of awe. This view, however, is open to the same worries raised against option C, since it also hinges on the alleged idea that ES is mostly a negative experience.

Option D can be supported by other means, pivoting on different ways of sorting out awe species. In the philosophical literature, Quinn (1997), for instance, distinguishes aesthetic and religious awe (see also Clewis, 2019). On this suggestion, option D is then the additional view that ES coincides with the former species of awe. Indeed, Quinn argues that ES is awe in the absence of religious belief. Among psychologists, Konečni (2011) seems to hold a similar view. Indeed, his aesthetic theory, which posits aesthetic awe as the peak aesthetic experience, treats the latter “as the prototypical subjective reaction to a *sublime stimulus-in-context*, (…) one aspect of aesthetic awe, which distinguishes it from awe that is induced by fear, is *existential security* of the experiencing person” (p. 65). There are no *prima facie* reasons against this reading of option D, which remains a workable option.

As far as we know, option E, according to which awe is a species of ES, has not been pursued in the psychological literature. In philosophy, it seems that only Burke (1759) has explicitly endorsed it. According to him, the “highest degree” of ES is *astonishment* and its “subordinate degrees” are *awe*, *reverence*, and *respect* (p. 123). One way of supporting E is to appeal to a distinction drawn by Shapshay (2013a, b) between two varieties of ES: while the “thin sublime” is a largely non-cognitive, affective arousal, the “thick sublime” also involves a cognitive play of ideas (especially about the place of human beings within the environment). On the hypothesis that awe is a purely non-cognitive, emotional response (but see fn1), it might be suggested that it coincides with a species of ES, namely the “thin sublime.”

The question is not settled, however, since the idea that awe is an emotion can also lead to option D. If awe is a basic emotion and the sublime is a culturally specific category (as suggested in the classic study by Nicolson, 1963), then awe may very well be an ingredient of ES, but it would unlikely be the other way around.

### Only a Common Denominator Between Awe and the Experience of the Sublime

Finally, according to option F, there is a common denominator between awe and ES, although they differ from each other in all other respects. Building on our previous discussion, a plausible suggestion is that they involve the same kind of *negative* affective appraisal. Both experiences involve being overwhelmed by a stimulus too vast (big, powerful, etc.) for our ordinary cognitive ways of apprehending and coping with the world. Option F goes further, and states that awe and ES do not have anything else in common. In particular, they involve different kinds of *positive* affective evaluation (or only ES has an overall positive valence after all).

## Discussion

What should we conclude from the foregoing critical comments on the complex relationship between awe and ES? Let’s start by taking on board the plausible suggestion just made, that they involve at least the same kind of negative affective appraisal. Now both awe and ES also involve a positive evaluation. They are the kind of experience that we seek for and want to reproduce. The next question is then whether we should consider the positive evaluation involved in awe to be also involved in ES, and how.

Suppose, as is sometimes claimed (see Brady’s quotation in the previous section), that the positive evaluation in awe is *admiration*. If this positive evaluation is also involved in ES, it follows that in having the latter, aesthetic experience, we experience admiration. Now what would be the object of our admiration? In the case of religious awe, it is obvious what the object of admiration is, namely God (or some divinity). In contrast (although the point is certainly controversial), it is not all obvious that admiration is the key concept involved in ES, or more generally in aesthetic experience.

An interesting proposal, put forward by McShane (2013), is that awe involves an evaluation of the *importance* of the awe-inspiring object, which *impresses* us in some respect. This might lead to a defense of option D: ES would be a species of awe, namely aesthetic awe. This defense would go like this. The concept of importance is relatively formal, and there are different types of importance. Thus, if all cases of awe involve the same *kind* of positive evaluation (the object of awe is subjectively evaluated as being of great importance), different cases of awe concern different *species* of importance. One of these species is aesthetic importance, or importance from an aesthetic point of view. Of course, such a defense should make clear what aesthetic importance is precisely, but it would be a way of reconciling two (apparently conflicting) intuitions we might have about awe and ES, that they are very close experiences, and that awe need not be an aesthetic experience.

We suspect that any psychological study of awe, whatever its valence (positive or negative) and the domain it concerns (aesthetic, religious, social, etc.) should take a stance on its relationship with ES. At the same time, though, philosophers should get more interested in awe itself and its role in the determination of the overall valence of ES. What we have offered here is of course only an exercise in conceptual geography, and further interdisciplinary studies should go deeper in the specification of, and comparison among, the more promising options we have delineated here.

## Author Contributions

MA, MS, AJ, PP, and JD have contributed equally to the analysis of the literature and to the finalization of the manuscript. MA proposed the structure of the presentation (with JD) and has been the main redactor.

## Conflict of Interest

The authors declare that the research was conducted in the absence of any commercial or financial relationships that could be construed as a potential conflict of interest.

## Footnotes

1 Awe is almost always considered to be an emotion or an emotional construct. Whether it represents a single construct referred to by all researchers is of course debatable. We suspect that clarifying its relationship to the experience of the sublime will also clarify its nature. Another issue concerns the relationship between emotion and cognition. When we consider awe as an emotion, we leave it open whether it also involves cognitive elements.

## Abbreviations

ES: experience of the sublime.
